# Occult Hypercalcemia Unmasked: Insights From Remitting Seronegative Symmetrical Synovitis With Pitting Edema (RS3PE) Syndrome Associated With a Gastrointestinal Lymphoma

**DOI:** 10.7759/cureus.95228

**Published:** 2025-10-23

**Authors:** Zhihan Xiang, Yuying Yang, Weiwei Rui, Jialin Teng, Yutong Su

**Affiliations:** 1 Department of Rheumatology and Immunology, Ruijin Hospital, Shanghai Jiao Tong University School of Medicine, Shanghai, CHN; 2 Department of Endocrine and Metabolic Diseases, Ruijin Hospital, Shanghai Jiao Tong University School of Medicine, Shanghai, CHN; 3 Department of Pathology, Ruijin Hospital, Shanghai Jiao Tong University School of Medicine, Shanghai, CHN; 4 Department of Rheumatology and Immunology, Shanghai Hospital of Civil Aviation Administration of China, Shanghai, CHN

**Keywords:** diffuse large b-cell lymphoma, hypercalcemia, paraneoplastic syndrome, parathyroid hormone-related protein (pthrp), rs3pe syndrome

## Abstract

Remitting seronegative symmetrical synovitis with pitting edema (RS3PE) is a rare inflammatory arthritis that may present as a paraneoplastic syndrome. We report a case of a 51-year-old woman who initially responded to high-dose steroids for RS3PE but later developed persistent hypercalcemia. Serum calcium ranged from 3.03 to 4.02 mmol/L, with suppressed parathyroid hormone (PTH), suggesting a non-parathyroid cause. Despite initial suspicion of infection-related hypercalcemia, calcium levels remained elevated after antimicrobial therapy. The 25-hydroxyvitamin D level was elevated, although serum 1,25-dihydroxyvitamin D was not directly measured. Immunohistochemistry revealed strong expression of parathyroid hormone-related protein (PTHrP) and 25-hydroxyvitamin D3-1α-hydroxylase (CYP27B1), indicating potential contributions from both humoral and vitamin D-mediated pathways. The patient received supportive treatment with diuretics, calcitonin, and denosumab, which successfully corrected hypercalcemia. Gastrointestinal endoscopy and biopsy ultimately confirmed diffuse large B-cell lymphoma (DLBCL). She then underwent anti-lymphoma chemotherapy, resulting in sustained remission and stable calcium levels. This case highlights the diagnostic challenges of RS3PE with hypercalcemia and the importance of considering underlying malignancy when standard therapy fails. It also demonstrates that PET-CT may fail to detect tumors in hollow organs and that gastrointestinal endoscopy should be considered in unresolved cases. Early recognition of tumor-associated hypercalcemia and a mechanism-based diagnostic approach are essential for effective treatment.

## Introduction

Remitting seronegative symmetrical synovitis with pitting edema (RS3PE) is a rare rheumatologic syndrome first described in 1985, characterized by acute onset, symmetrical joint swelling, and pitting edema in the limbs [1]. Most patients respond well to glucocorticoids. It is seen primarily in older adult men. Although the exact incidence is unknown, a retrospective study of 6,868 patients attending a primary care clinic in Japan identified RS3PE syndrome in three patients (0.04%), with an average age at diagnosis of 69 [2]. Its rarity, combined with clinical features that overlap with polymyalgia rheumatica, seronegative rheumatoid arthritis, and paraneoplastic rheumatic syndromes, poses a significant diagnostic challenge. Moreover, RS3PE may occasionally be associated with underlying malignancies, particularly hematologic cancers, which are often difficult to detect early.

This paper reports a case of RS3PE in a female patient accompanied by refractory hypercalcemia, who was ultimately diagnosed with aggressive diffuse large B-cell lymphoma (DLBCL).

## Case presentation

A 51-year-old woman presented with unexplained swelling and pain in the hands, wrists, shoulders, knees, and ankles, accompanied by pitting edema in the distal extremities. She reported significant tenderness, morning stiffness, and limited range of motion (especially in the arm and elbow). There was no family history of genetic disorders or chronic diseases. On examination, pitting edema was observed over the hands and feet. Tenderness and mild swelling were noted in the wrists, shoulders, elbows, knees, and ankles. Palpable lymphadenopathy was present in the axillary regions bilaterally.

**Figure 1:**
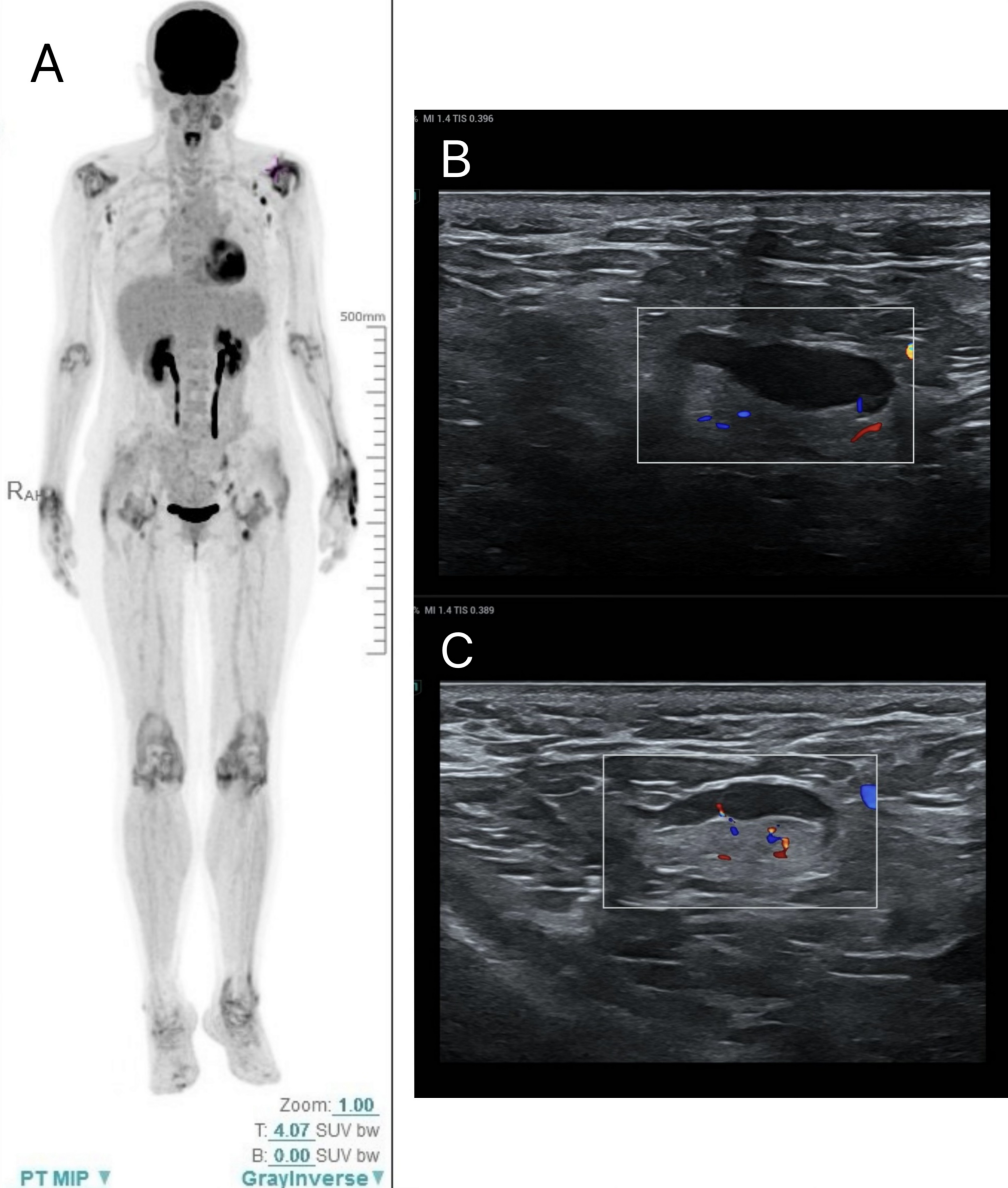
PET-CT showing diffusely increased metabolic activity in the periarticular soft tissues of the extremities and axillary lymph node ultrasound A: After fasting for over six hours and an intravenous injection of ¹⁸F-FDG, diffuse metabolic uptake is observed in the periarticular soft tissues of bilateral wrists, elbows, shoulders, hips, knees, and ankles (SUVmax ~5.5). B-C: Ultrasound demonstrates enlarged bilateral axillary lymph nodes.

Laboratory investigations revealed elevated inflammatory markers, including C-reactive protein (CRP: 83 mg/L; normal: ≤5.0 mg/L), erythrocyte sedimentation rate (ESR: 106 mm/h; normal: ≤20 mm/h), and D-dimer (4.29 mg/L; normal: ≤0.5 mg/L), indicating a high inflammatory state. Rheumatoid factor, antinuclear antibody (ANA), anti-CCP, anti-Sm, SSA, SSB, and anti-dsDNA antibodies were all negative. Bone marrow aspiration revealed active, but unevenly distributed, granulocytic, erythroid, and megakaryocytic proliferation, with a decreased granulocyte-to-erythroid ratio. Axillary lymph node ultrasound showed enlarged axillary lymph nodes, but biopsy was not feasible due to pain and limited arm mobility (Figures 1B, 1C). PET-CT showed diffusely increased metabolic activity in the soft tissues of limb joints, without signs of malignancy (Figure 1A). Based on clinical symptoms and laboratory examinations, the diagnosis of RS3PE was made. The patient received intravenous methylprednisolone (80 mg), combined with tofacitinib and methotrexate, and was supplemented with calcium and gastric protection. The symptoms improved quickly, with CRP decreasing to 12 mg/L and ESR to 22 mm/h. She was discharged with a tapered steroid regimen.

**Figure 2:**
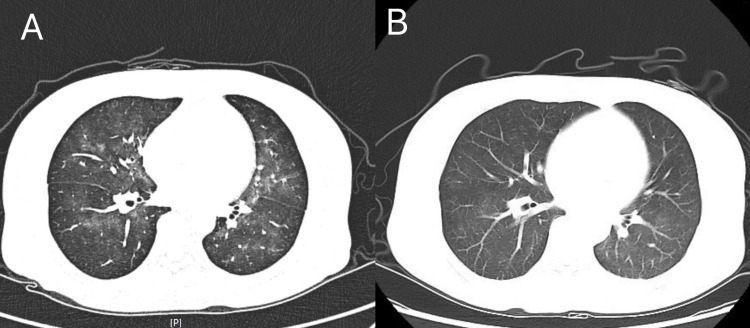
Chest CT (A) before and (B) after treatment A: Scattered patchy ground-glass opacities in both lungs, consistent with exudative lesions. B: Partial resolution of the exudative lesions after treatment.

Four months later, the patient presented again, reporting fatigue, poor appetite, and accompanying nausea and vomiting. Additionally, the patient presented a persistent dry cough and fever, reaching up to 38.5°C, associated with night sweats but no chills or rigors. Serum calcium was elevated at 3.03 mmol/L. Infection-related biomarkers, including procalcitonin (PCT), endotoxin, β-1,3-D-glucan, and T-SPOT, showed no abnormalities. Chest CT revealed scattered ground-glass opacities in both lungs (Figure 2A). Next-generation sequencing (NGS) of peripheral blood detected *Pneumocystis jirovecii*, suggesting *Pneumocystis jirovecii* pneumonia (PJP) infection. The patient was treated with the intravenous compound sulfamethoxazole (4 mL, twice daily for eight days). Her coughing improved, her body temperature normalized, and a follow-up chest CT showed partial absorption of the pulmonary infiltrates (Figure 2B). Previous reports indicated that PJP infection could lead to elevated serum calcium levels. However, the patient's hypercalcemia did not improve after antimicrobial therapy (serum calcium: 3.32 mmol/L).

As shown in the day-by-day timeline in Supplementary Table 1, the patient's serum calcium continued to increase progressively. She was treated with intravenous torasemide (10 mg) for diuresis and calcitonin (salmon) at 50 U every 12 hours for three days and every eight hours for another three days. However, the effect was poor, with serum calcium peaking at 4.02 mmol/L. The dose of calcitonin was increased to 100 IU every eight hours, and subcutaneous injections of denosumab (120 mg on day one and day eight) were administered, alongside intravenous normal saline (4000-6000 mL/day) to correct mild volume depletion. Laboratory evaluation revealed low-normal parathyroid hormone (15.1 pg/mL), elevated serum phosphate (2.05 mmol/L), elevated 25-hydroxyvitamin D (54.49 ng/mL), and serum albumin of 33 g/L, yielding an albumin-corrected calcium of 4.14 mmol/L. Urinary calcium excretion was 19.08 mg/kg/24 h.

At the time of collection, the patient had significant renal impairment (serum creatinine 232 μmol/L, urea 22.9 mmol/L), was mildly volume-depleted (intake 3335 mL, output 4400 mL) due to vomiting and inability to maintain oral intake, and urine was collected over 24 hours under usual dietary conditions. Given impaired renal clearance and negative fluid balance, urinary calcium may underestimate true calciuria. Despite these limitations, the combination of low-normal PTH, elevated phosphate, elevated 25-OH vitamin D, and positive CYP27B1 immunohistochemistry supports a diagnosis of non-PTH-mediated hypercalcemia, guiding further investigation and management.

**Figure 3:**
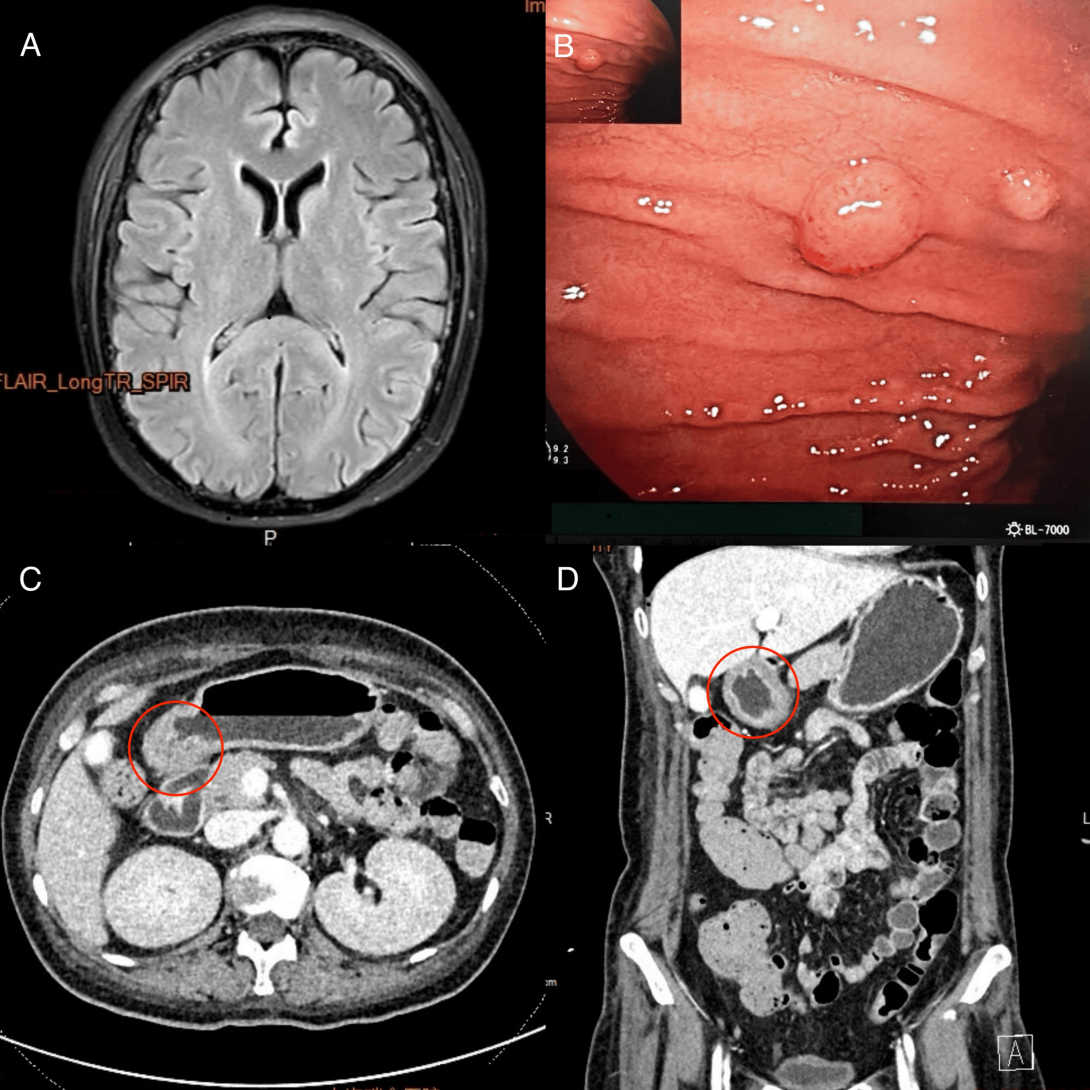
The MRI image of the brain, gastroscopy, and contrast-enhanced CT of the small intestine A: Brain MRI (without contrast) showing no significant abnormalities and no meningeal signs. B: Gastric body and fundus: smooth mucosal folds. A polypoid lesion measuring approximately 1 cm was observed on the greater curvature near the anterior wall of the mid-body, along with multiple broad-based polyps. C-D: CT of the small intestine (contrast-enhanced, non-ionic contrast agent (100 mL) was injected via the antecubital vein at 3 mL/s) after oral mannitol showed thickening of the gastric antrum with a layered hypodense area in the submucosa.

Considering that RS3PE is often associated with malignancies, tumor-related hypercalcemia was highly suspected. Repeat bone marrow aspiration showed mildly to moderately decreased trilineage hematopoiesis, with a small number of scattered or small clusters of lymphocytes accounting for less than 5% of nucleated cells. In order to identify the causes of the patient's nausea and vomiting, a brain MRI was taken, which excluded central causes of vomiting (Figure 3A). Enhanced CT of the small intestine revealed thickening of the gastric antrum with abnormal mucosal enhancement (Figures 3C, 3D). Gastroscopy showed focal proliferative lesions on the anterior wall of the gastric body and multiple polyps from the gastric fundus to the body, including a large protruding lesion and several small polyps (Figure 3B). Histopathological examination showed diffuse infiltration of atypical lymphocytes. Immunohistochemistry revealed positive expression of CD20, BCL-6, BCL-2, and MUM-1 in tumor cells. Molecular studies confirmed clonal rearrangement of immunoglobulin (IG) genes. The final pathological diagnosis was DLBCL.

**Figure 4:**
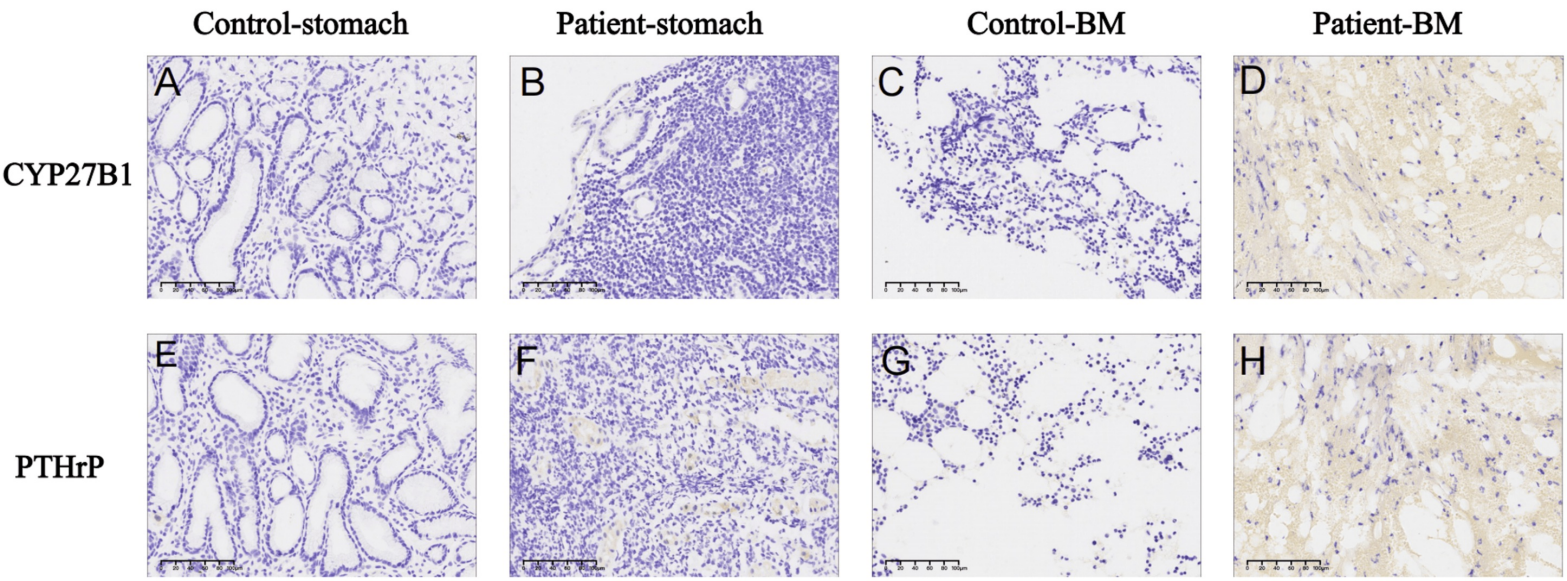
The IHC staining of CYP27B1 and PTHrP in gastric and bone marrow tissues A–D: Immunohistochemical (IHC) staining results show that CYP27B1 expression is nearly absent in the control group's gastric tissue (Control-stomach, A) and bone marrow (Control-BM, C). In contrast, specific CYP27B1 expression (yellow) is detected in the patient's gastric tissue (Patient-stomach, B) and bone marrow (Patient-BM, D) on serial sections. E–H: PTHrP expression shows only mild background staining in fibrous tissue of the control group (E, G), whereas specific PTHrP expression is observed in the patient's gastric tissue (Patient-stomach, F) and bone marrow (Patient-BM, H).

We further performed immunohistochemical (IHC) staining on the patient's gastric polyps and bone marrow biopsy specimens. Compared with control stomach and bone marrow tissues, the expression of 25-hydroxyvitamin D3-1α-hydroxylase (CYP27B1) (Figures 4A, 4C) and parathyroid hormone-related protein (PTHrP) (Figures 4E, 4G) was significantly increased in the patient's gastric and bone marrow tissues (Figures 4B, 4D for CYP27B1 and Figures 4F, 4H for PTHrP). These findings further confirmed that the hypercalcemia was caused by tumor secretion of PTHrP and CYP27B1. The patient was pathologically diagnosed with DLBCL and was transferred to the hematology department for further anti-lymphoma treatment. By November 24, 2024, she had completed three cycles of chemotherapy, and her general condition was stable.

## Discussion

RS3PE has been considered to be a paraneoplastic syndrome, with an association rate with malignancies ranging from 16% to 30% [3]. Among associated solid tumors, adenocarcinomas are most frequently reported, whereas hematologic malignancies include non-Hodgkin lymphoma, myelodysplastic syndrome, and chronic lymphocytic leukemia [4]. The underlying mechanisms are not fully understood but may involve tumor-secreted factors such as vascular endothelial growth factor (VEGF), interleukin-6 (IL-6), or PTHrP, which can promote synovial inflammation and edema, or cross-reactivity of tumor-targeted antibodies leading to immune-mediated joint involvement. Patients with paraneoplastic RS3PE often respond poorly to glucocorticoid therapy and typically require higher doses of prednisone to relieve such symptoms [5]. They may also present with systemic manifestations such as fever, anorexia, and weight loss. In some cases, RS3PE symptoms may improve after tumor resection.

In this case, the patient presented with distal joint pain and pitting edema of the limbs, along with elevated acute-phase reactant findings consistent with RS3PE. The patient exhibited a marked inflammatory state and gradually improved with high-dose prednisone. However, clinical signs of an occult gastric lymphoma emerged after four months of follow-up. Therefore, it is recommended that patients diagnosed with RS3PE undergo clinical evaluation and basic cancer screening every three months during the first year (especially those at high risk), followed by re-evaluation every six months during years two to three. If no malignancy is detected, the interval can be gradually extended, transitioning to annual follow-ups after the third year [6]. Additionally, this case highlights that PET-CT has limited sensitivity for the early detection of tumors in hollow organs such as the stomach, a finding also reported in other cases. Its sensitivity decreases when lesions are small (<30 mm) or of certain histological subtypes, such as signet-ring cell or mucinous carcinoma [7]. As a result, gastrointestinal endoscopy should be performed when clinically indicated to aid in diagnosis.

This patient presented with treatment-resistant RS3PE accompanied by hypercalcemia, necessitating a thorough differential diagnosis. Hypercalcemia can be broadly classified into parathyroid hormone (PTH)-dependent and PTH-independent types. The former is most commonly caused by primary hyperparathyroidism, characterized by elevated PTH levels and hypophosphatemia. The latter is most often associated with malignancies and is typically characterized by an acute onset (serum calcium often >3.5 mmol/L), suppressed or low-normal PTH levels, and a high risk of developing hypercalcemic crisis [8,9]. Humoral hypercalcemia mediated by PTHrP accounts for approximately 80% of cases and is the most common cause of malignancy-related hypercalcemia. Clinically, hypercalcemia may manifest as neuropsychiatric symptoms, gastrointestinal disturbances, and renal dysfunction [10,11]. Based on the underlying mechanisms, tumor-induced hypercalcemia can result from osteolytic bone destruction, excessive secretion of PTHrP, or increased activation of 1,25-dihydroxyvitamin D. These mechanisms are generally associated with suppressed PTH levels. Other differential considerations include granulomatous diseases (e.g., sarcoidosis, tuberculosis, infections), vitamin D-related disorders, hyperthyroidism, medication effects, FGF23-related disorders, and genetic conditions such as familial hypocalciuric hypercalcemia (FHH) [12].

In this case, the patient was initially diagnosed with PJP infection according to a series of symptoms and laboratory tests. PJP infection can also cause hypercalcemia, typically in association with granulomatous inflammation. Granulomas are formed by activated macrophages, which can produce 1-α-hydroxylase that converts 25-hydroxyvitamin D (25(OH)D) into its active form, 1,25-dihydroxyvitamin D₃ (1,25(OH)₂D₃). This active form enhances intestinal calcium absorption and bone resorption, leading to elevated serum calcium, while PTH levels are typically suppressed. Furthermore, the clinical features of PJP, such as fever, cough, and dyspnea, can mimic those of lymphoma, potentially masking tumor symptoms and delaying diagnosis [13,14]. In this case, although the patient's fever resolved and respiratory symptoms improved after antimicrobial treatment, her hypercalcemia persisted (calcium level: 4.02 mmol/L), suggesting that PJP was not the primary cause. Further investigation for malignancy was warranted.

Gastrointestinal evaluation was performed, and a gastric tissue biopsy under gastroscopy confirmed a diagnosis of DLBCL. Lymphoma cells can secrete PTHrP, which directly stimulates osteoclast activity and inhibits osteoblast function, resulting in net bone resorption. In addition, PTHrP enhances renal tubular calcium reabsorption, further contributing to elevated calcium levels [15].

A second, distinct mechanism involves dysregulated production of 1,25-dihydroxyvitamin D. Tumor cells and tumor-associated macrophages may aberrantly express 25-hydroxyvitamin D3-1α-hydroxylase (CYP27B1), which is not suppressed by PTH or serum calcium. This enzyme converts 25(OH)D into the biologically active 1,25(OH)₂D₃, thereby increasing intestinal calcium absorption [16]. While serum 1,25-dihydroxyvitamin D was not directly assessed, elevated 25(OH)D levels provided abundant substrate for conversion. Immunohistochemistry revealed strong CYP27B1 expression in gastric tissue, supporting dysregulated local activation of vitamin D as a mechanism of hypercalcemia. Importantly, 25(OH)D elevation alone does not cause hypercalcemia; rather, in the context of CYP27B1 overexpression, it suggests enhanced production of active 1,25(OH)₂D. Moreover, inflammatory cytokines within the lymphoma microenvironment can further upregulate CYP27B1 expression in macrophages, amplifying 1,25(OH)₂D₃ production [17].

Together, these findings suggest that both PTHrP-mediated humoral hypercalcemia and CYP27B1-driven vitamin D activation contributed to the patient's refractory hypercalcemia.

In the treatment of tumor-related hypercalcemia, emergency calcium reduction is crucial during a hypercalcemic crisis. The first step is to restore blood volume through rehydration with 0.9% normal saline, followed by administration of calcitonin and the use of bisphosphonates (or denosumab) to suppress bone resorption [18]. In this patient, due to concomitant heart failure, the volume of intravenous fluids was restricted. With poor appetite and vomiting, parenteral nutrition was initiated, which restricted normal saline infusion and led to ineffective calcium reduction. Subsequently, treatment with denosumab and calcitonin was administered, alongside reduced parenteral nutrition, increased saline and water intake, and careful monitoring of fluid balance to correct dehydration. Serum calcium gradually normalized. During the acute management of tumor-related hypercalcemia, in addition to the timely use of antiresorptive agents and regular monitoring of serum calcium (every 24-48 hours), cardiac, and renal function [19], maintaining fluid-electrolyte balance and ensuring adequate hydration are also essential.

## Conclusions

RS3PE is considered a paraneoplastic syndrome, and patients require careful tumor screening during both diagnosis and follow-up. Clinicians should be alert to key red flags (poor steroid response, systemic symptoms, refractory hypercalcemia, unexplained weight loss and persistent fever), obtain a targeted laboratory panel (PTH, phosphate, 25(OH)D, 1,25(OH)₂D, PTHrP), and follow an imaging cascade, escalating from PET-CT to endoscopy if indicated by clinical or PET-CT findings suggestive of malignancy. Early oncology referral and structured follow-up are essential for detecting occult tumors and optimizing outcomes.

This case highlights the diagnostic challenges of RS3PE when linked with refractory hypercalcemia and stresses the need for thorough malignancy screening in atypical or treatment-resistant cases. PET-CT missed the gastric lymphoma in this case, showing the limits of metabolic imaging in hollow organs; endoscopy and biopsy were crucial for diagnosis. Tumor-induced hypercalcemia here involved multiple mechanisms, including PTHrP overproduction and extrarenal vitamin D activation by tumor cells. These should be considered in cases of severe or persistent hypercalcemia when common causes have been ruled out. Prompt recognition of hypercalcemia, use of antiresorptive therapy, and fluid balance were key to the management of hypercalcemia. This case underscores the importance of a multidisciplinary, mechanism-focused approach and recommends regular malignancy screening, including gastrointestinal evaluation, during RS3PE follow-up.

## Appendices

**Table 1:** Detailed day-by-day clinical timeline showing steroid regimen, disease-modifying treatments, laboratory parameters, and symptom evolution Timeline summarizing key laboratory parameters (creatinine, urea, albumin, total protein, calcium, phosphate, 25-OH-Vitamin D, PTH, CRP, ESR), corticosteroid dose and taper, introduction or withdrawal of disease-modifying therapies (tofacitinib, methotrexate, hydroxychloroquine), calcium-lowering agents (calcitonin, denosumab), and corresponding clinical symptoms. The values separated by a slash (/) represent two laboratory measurements obtained at different times on the same day. IV = intravenous; PO = oral; SC = subcutaneous; qd = once daily; bid = twice daily; qw = once weekly.

Date	Creatinine (μmol/L)	Urea (mmol/L)	Albumin (g/L)	Total Protein (g/L)	Serum Calcium (mmol/L)	Serum Phosphate (mmol/L)	Corrected Calcium (mmol/L)	25-OH-Vitamin D (nmol/L)	PTH (pg/mL)	CRP (mg/L)	ESR (mm/h)	Calcium-Lowering Medication	Primary Disease Treatment	Clinical Symptoms
Reference Range	53–97	2.5–7.1	35–55	60–83	2.00–2.52	0.85–1.51	2.11–2.52	≥50	15.0–68.3	<10	F: 0–20 M: 0–15	-	-	-
The first hospitalization														
4.9	48	2.5	31	68	2.18	1.15	2.36	47.10	-	83	66	-	-	-
4.10–4.14	-	-	-	-	-	-	-	-	-	-	-	-	Methylprednisolone 80 mg IV qd	Marked swelling and pain in the PIP joints of both hands, wrists, shoulders, knees, and ankles, with moderate edema of both upper and lower limbs.
4.15	57	4.0	27	58	2.26	1.12	2.50	-	-	12	22	-	Methylpredni-solone 80mg IV qd, Tofacitinib 5mg PO bid, Methotrexate 10mg PO qw, Hydroxychloroquine roquine 200mg PO bid	
4.16	-	-	-	-	-	-	-	-	-	-	-	-	Methylprednisolone 60 mg IV qd, Tofacitinib 5 mg PO bid, Methotrexate 10mg PO qw, Hydroxychloroquine 200 mg PO bid	Swelling and pain in the PIP joints of both hands, wrists, shoulders, knees, and ankles improved, along with mild edema in both lower limbs.
4.17	Discharge Oral medications: Prednisone 50 mg qd, Tofacitinib 5 mg bid, Methotrexate 10 mg qw, and Hydroxychloroquine 200 mg bid mild edema of both lower limbs, accompanied by improved swelling and pain in the wrists, shoulders, knees, and ankles. The patient was discharged with outpatient follow-up and gradual tapering of corticosteroids.													
The second Hospitalization														
6.24	92	6.3	28	59	3.10	1.12	3.24	67.43	-	55	45	-	Prednisone 15 mg PO qd, Hydroxychloroquine 200 mg PO bid (Considering infection, methotrexate and Tofacitinib were discontinued in the outpatient setting)	Significant pain in the left buttock and both lower limbs, with mild edema in the left lower limb.
6.25	-	-	-	-	-	-	-	-	-	-	-	-		
6.26	114	5.4	28	55	3.03	1.01	3.39	-	-	53	52	-	Methylpredni-solone 20 mg IV qd (Administered intravenously due to nausea and vomiting, making oral medications difficult)	
6.27	121	6.3	28	52	3.22	1.08	3.46	-	-	57.8	-	-		
6.28	142	8.2	29	54	3.25	0.88	3.43	-	6.5	66	55	-		
6.29	133	7.0	30	58	3.32	0.97	3.48	-	-	48	-	Calcitonin (Salmon) 50U SC q12h		
6.30	139	9.2	30	62	3.17	0.92	3.33	-	-	29	-			
7.2	217	15.2	28	66	3.14	1.20	3.28	-	-	14	68			
7.3	203	15.4	31	69	3.25	1.04	3.43	-	-	10	-	Calcitonin (Salmon) 50U SC, q8h		
7.4	201	20.7	37	72	3.73/3.59	1.66/1.64	3.79	-	-	10	63			
7.5	216	21.3	32	64	3.88	1.6	3.96	-	-	27	-	Denosumab 120 mg SC once, Calcitonin (Salmon) 50U SC q8h	Methylprednisolone 40 mg IV qd (due to fatigue, anorexia, worsening joint pain, and increased bilateral lower limb edema compared with previous assessment)	Worsening bilateral lower limb pain with difficulty walking and increased facial and bilateral lower limb edema.
7.6	232	22.9	33	67	4.02/3.60	2.05/1.91	4.14/3.72	54.49	15.1	31	-	Calcitonin (Salmon) 100U SC q8h		
7.7	249	24.4	27	55	3.57/3.36	2.13/2.16	3.81/3.60	-	-	22	-			
7.8	311	25.9	27	52	3.19/2.94	2.39/2.14	3.43/3.18	-	-	20	-			
7.9	320	25.6	25	49	2.81/2.60	2.24/1.90	3.11/2.90	-	-	23	48	Calcitonin (Salmon) discontinued		
7.10	-	-	-	-	2.65	1.65	-	34.91	11.6	-	-	-		
7.11	272	19.1	25	45	2.55	1.23	2.85	-	-	13	-	-		
7.12	230	17.1	25	47	2.46	1.27	2.76	-	-	18	-	Denosumab 120 mg SC once		Slight improvement in bilateral lower limb pain, with mild facial and bilateral lower limb edema.
7.13	211	14.4	28	50	2.45	0.82	2.69	-	-	18	-	-		
7.14	201	12.9	26	45	2.20	0.90	2.48	-	-	37	-	-		
7.15	189	11.6	27	47	2.12	0.90	2.36	-	-	34	12	-		
7.16	173	10	30	48	2.05	0.80	-	-	-	28	-	-		
7.17	157	9.6	28	44	1.93	0.73	2.17	-	-	28	-	-		
7.18	141	8.4	29	46	1.96	0.94	2.14	-	-	31	10	-		
7.19	129	7.7	30	47	2.05	0.67	2.21	-	42.1	25	-	-	Dexamethasone 15 mg IV qd (Prednisone discontinued and switched to dexamethasone due to fever and concern for lymphoma after bone marrow aspiration)	
7.20	130	8.3	31	49	2.02	0.87	2.20	-	-	38	-	-		
7.21	-	-	-	-	-	-	-	-	-	-	-	-		
7.22	107	7.8	36	54	1.96	0.69	2.04	-	-	20	-	-		
7.23	115	7.9	33	48	1.93	0.75	2.07	-	-	24	6	-		
7.24	121	9.1	31	48	2.00	0.88	2.18	-	-	35	-	-	Dexamethasone reduced to 10mg IV qd	Improvement in bilateral lower limb pain and decreased facial and bilateral lower limb edema.
7.25	-	-	-	-	-	-	-	-	-	34	12	-		
Follow-up	The patient was further referred to the Hematology Department.													
